# A common multi-host parasite shows genetic structuring at the host species and population levels

**DOI:** 10.1017/S0031182024000428

**Published:** 2024-05

**Authors:** Clara L. Shaw, Rebecca Bilich, Meghan A. Duffy

**Affiliations:** 1 Department of Ecology & Evolutionary Biology, University of Michigan, Ann Arbor, MI, USA; 2 Department of Biology, University of Minnesota Duluth, Duluth, MN, USA

**Keywords:** *Daphnia*, genetic structure, *Pasteuria ramosa*, VNTR

## Abstract

Although individual parasite species commonly infect many populations across physical space as well as multiple host species, the extent to which parasites traverse physical and phylogenetic distances is unclear. Population genetic analyses of parasite populations can reveal how parasites move across space or between host species, including helping assess whether a parasite is more likely to infect a different host species in the same location or the same host species in a different location. Identifying these transmission barriers could be exploited for effective disease control. Here, we analysed population genetic structuring of the parasite *Pasteuria ramosa* in daphniid host species from different lakes. Outbreaks occurred most often in the common host species *Daphnia dentifera* and *Daphnia retrocurva*. The genetic distance between parasite samples tended to be smaller when samples were collected from the same lake, the same host species and closer in time. Within lakes, the parasite showed structure by host species and sampling date; within a host species, the parasite showed structure by lake and sampling date. However, despite this structuring, we found the same parasite genotype infecting closely related host species, and we sometimes found the same genotype in nearby lakes. Thus, *P. ramosa* experiences challenges infecting different host species and moving between populations, but doing so is possible. In addition, the structuring by sampling date indicates potential adaptation to or coevolution with host populations and supports prior findings that parasite population structure is dynamic during outbreaks.

## Graphical abstract

**Figure:**
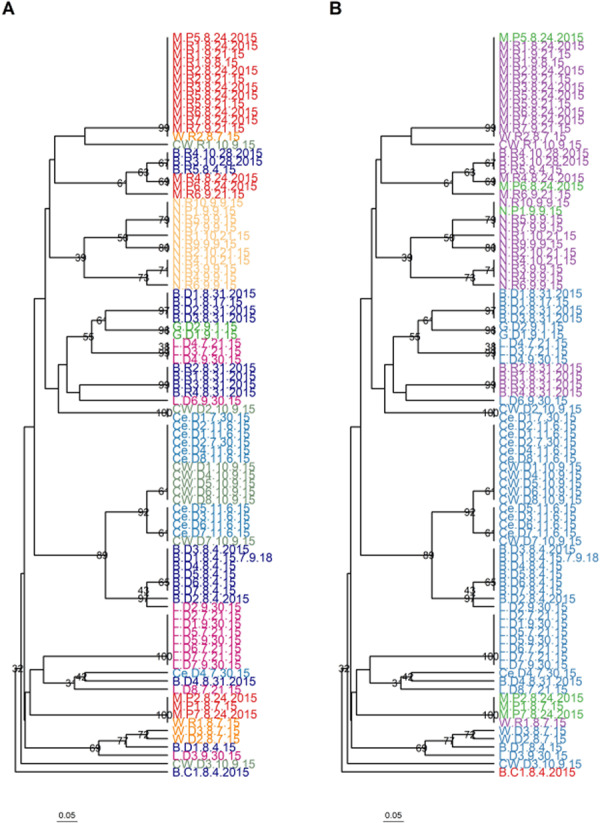

## Introduction

Parasite infection is ubiquitous, but the extent to which individuals and populations are impacted varies depending on a suite of genetic, seasonal, population-level, community-level and environmental factors (Betts *et al*., 2016). Given this complexity, it is often not clear from where invading parasites originate and how they get a foothold in a population. Parasites could invade a host population through transmission from an environmental reservoir (Decaestecker *et al*., 2004), from other infected populations (Penczykowski *et al*., 2016) or spillover from other host species in the community (Craft *et al*., 2009). Most likely, parasites come from all these (and other) pathways, but the relative contributions of transmission from a reservoir, from physically distinct host populations or across species barriers are unclear. Improving our understanding of parasite origins could help to predict outbreaks and to identify transmission barriers that could be useful in their prevention.

Each of these transmission routes has been explored in the ecological literature, but often not concurrently with other pathways. For example, parasite transmission can occur from an environmental spore bank where transmission stages remain viable without a host and later infect a non-contemporary host population (Dragon and Rennie, 1995). Such transmission through time could facilitate increased diversity and coexistence of competitors as has been demonstrated in other systems with storage effects (Cáceres, 1997). Therefore, if transmission occurs primarily from environmental spore banks, we expect high parasite diversity within populations and genetic structure between them if founder parasites from spore banks vary over space.

Alternatively, parasite transmission could occur from one host population to another of the same host species across a landscape. In these cases, dispersal between host populations depends on the parasite's ability to reach the new population, colonize local host genotypes, survive in a potentially different microclimate and compete with local parasite genotypes (Penczykowski *et al*., 2016; Ekholm *et al*., 2017). If parasite populations show strong spatial structure and low diversity, this could reflect challenges for parasites to overcome these barriers.

A third source of outbreaks is the transmission of parasites between host species within a community; indeed, these events have caused some of the most devastating epidemics because parasite virulence can be maladapted in novel hosts, and parasite production in reservoir host species is decoupled from novel host density (Daszak *et al*., 2000). Most parasites can infect multiple host species (Cleaveland *et al*., 2001), and studying transmission across host species may provide insight into factors that constrain cross-species transmission. An obvious challenge for parasites is that hosts may vary in susceptibility, competence and defences, imposing different selective forces on parasites (Gandon, 2004). However, phylogenetic relatedness of hosts and similarity of within-host environments may lower barriers to transmission (Streicker *et al*., 2010; Longdon *et al*., 2011; Parker *et al*., 2015; Shaw and Kennedy, 2022). The genetic structure of parasites across host species implicates patterns of multi-host parasite transmission in the wild: structure at the level of host species indicates barriers to transmission among hosts whereas lack of structure indicates frequent transmission between host species (Archie and Ezenwa, 2011).

Importantly, outbreaks might incorporate interacting aspects of transmission from the environment (and across time), over space, among host species, as well as evolutionary change. For example, in many emerging outbreaks, pathogens spill over from co-occurring host species and then evolve to exploit the new host (Fenner and Fantini, 1999; Delaney *et al*., 2012). In addition, pathogens may be introduced from other populations and then evolve to exploit hosts in a new environment (Burdon and Thrall, 1999; Koskella, 2014). Moreover, multiple mechanisms might occur at once. For example, after its introduction to the United States, West Nile virus evolved to transmit more efficiently in new mosquito vectors and also spread in mosquito and vertebrate populations across the continent (Kilpatrick, 2011). By analysing parasite population structures, we can learn about the relative influences of these different processes, which can provide us a better understanding of how parasites transmit in complex environments.

Here, we studied the population structure of *Pasteuria ramosa*, a wide-spread parasite of daphniid hosts, which are important planktonic grazers in freshwater systems (Ebert, 2008). *Pasteuria ramosa* is a genotypic specialist within host species due to specific interactions between the host and parasite that govern attachment of spores to cells in the host oesophagus after spores are consumed during feeding (Duneau *et al*., 2011; Routtu and Ebert, 2015; Bento *et al*., 2017) as well as additional within-host processes (Luijckx *et al*., 2014). Infection of two host species by the same parasite ‘variety’ has been reported to be a rare event (Duneau *et al*., 2011; Luijckx *et al*., 2014) though one experiment passaged *P. ramosa* from one host species, through a second, then back to the first (Auld *et al*., 2017). After infection, the parasite castrates its host and propagates itself within the host haemolymph (Ebert *et al*., 1996). *Pasteuria ramosa* is an obligate killer, and spores are only released from decaying host corpses (Ebert *et al*., 1996). These spores can remain infective for many decades in lake sediments (Decaestecker *et al*., 2004, 2007). It is possible that *P. ramosa* varieties specializing on different hosts are in fact distinct species. However, since the research community uses ‘*Pasteuria ramosa*’ to refer broadly to this parasite that infects across daphniid hosts, we have chosen to do so as well, and to use the term ‘genotype’ to refer to samples with distinct molecular signatures.

We investigated whether *P. ramosa* outbreaks were genetically distinct among lakes, if *P. ramosa* genotypes commonly moved between host species and if *P. ramosa* genetic structure changed over time using analyses of variable number tandem repeat (VNTR) data. We predicted that populations of this parasite would be differentiated by lake since we predicted that transmission from spore banks would be common, transmission between lakes would be low and selection within lakes would be strong. Similarly, we predicted that the parasite would not move readily between host species given previous findings of host genotype specificity. Finally, we predicted that parasite genetic structure would change over time given changing host population structure and environmental factors through an epidemic season.

## Materials and methods

### Field sampling

We sampled eight lakes (Table 1) in southeastern Michigan every two weeks from mid-July until mid-November 2015. On each date, we combined three vertical plankton tows of the whole water column (collected using a 12 cm diameter Wisconsin net with 153 μm mesh); the three tows were collected from at least 10 m apart within the deep basin of each lake. We then used random subsamples of these tows to assess the prevalence of *P. ramosa* infection; all daphniids (*Daphnia dentifera*, *Daphnia retrocurva*, *Daphnia parvula*, *Daphnia pulicaria*, *Daphnia ambigua*, *Daphnia dubia*, *Ceriodaphnia dubia*) were counted and diagnosed for *P. ramosa* infection. This was performed using a dissecting microscope (25–50× magnification), counting and diagnosing at least 200 hosts of each species or, when hosts were rare, until the entire sample was processed. The earliest stages of infection are not apparent (Ebert *et al*., 1996), so we were only able to diagnose later-stage infections. We assessed host density using a second sample that also combined three vertical tows from the same locations. The density sample was initially preserved in 90% ethanol and later subsampled to determine host density. We multiplied host density and prevalence of infection on a given date to determine infected host density. We did not perform statistical analyses on the field data, but epidemics were visualized using ggplot2 version 3.4.0 (Wickham, 2016) in R version 4.2.2 (R Core Team, 2020).

**Table 1.** Lake names and locations

Lake	Michigan township	Lat., long.
Bishop	Hamburg Township	42.501259, −83.839804
Cedar	Sylvan Township	42.314426, −84.077480
Crooked (W)	Sylvan Township	42.326613, −84.111816
Gosling	Putnam Township	42.439565, −84.003322
Little Appleton	Hamburg Township	42.506705, −83.838634
Mill	Sylvan Township	42.329787, −84.090868
North	Dexter Township	42.393928, −84.006628
Walsh	Sylvan Township	42.337922, −84.080098

### Genotyping *P. ramosa*

We collected infected hosts from the field samples while processing the live sample to determine prevalence of infection. These were preserved individually in 90% ethanol at −20°C until DNA extraction. A mericon bacteria plus DNA extraction kit (Qiagen, Hilden, Germany) was used to extract DNA from 103 infected animals. Briefly, we removed the preserved infected animals from ethanol and placed them in sterile microcentrifuge tubes. We then vortexed them with a battery-powered pestle in 200 μL fast lysis buffer. After the sample was emulsified, we transferred it to bead tubes. These tubes were vortexed for 10 min, then centrifuged and then the DNA-containing supernatant was removed and preserved. The extracted samples were amplified at 11 VNTR loci (Mouton and Ebert, 2008; Andras and Ebert, 2013; Table 2) by polymerase chain reactions (PCRs). For these reactions, we used 5 μL Qiagen multiplex mastermix (QIAGEN, Hilden, Germany), 10 nm forward primer with M13(−21) tail, 400 nm reverse primer and 400 nm M13(−21) 6FAM labelled forward primer or M13(−21) HEX-labelled forward primer. The labelled primers allowed all loci to be visualized in fragment analysis (Schuelke, 2000). We used 2 μL of extracted DNA in total reaction volumes of 10 μL. We used the following amplification conditions: 94°C (15 min), then 42 cycles of 94°C (30 s)/50°C (30 s)/72°C (1 min) and a final extension time at 72°C for 10 min. After PCRs, we diluted 1 μL amplified product in 199 μL molecular-grade water. We then loaded 1 μL of this diluted product into prepared capillary electrophoresis loading plates containing 11 μL Hi-Di formamide and a LIZ500 (or a ROX500) size standard (University of Michigan DNA Sequencing Core). To visualize more samples on a single plate, both HEX and 6FAM dyes were used in some cases. In these cases, we diluted each of two distinctly labelled samples (1 μL each) in 198 μL molecular-grade water and then loaded 1 μL of the diluted combination into a well in the prepared capillary electrophoresis loading plates. The University of Michigan DNA Sequencing Core performed the fragment analysis; we used GeneMapper (Thermo Fisher Scientific, Waltham MA, USA) software to read fragment lengths.

**Table 2.** Forward and reverse primers used to genotype each locus

Locus	Forward (5′–3′)	Reverse (5′–3′)
Pr 1^a^	TGTAAAACGACGGCCAGTACCTAAAGAACAGGAATATCTGGA	GCATGGAATGATTTTTGCTG
Pr 2^a^	TGTAAAACGACGGCCAGTCTGCTGGATGGATGGACTACGTGA	ACCGGTCCCGTAGGTATAGG
Pr 3^a^	TGTAAAACGACGGCCAGTGGACCAATCGAACCAGGTAT	AACGGTTTCTTCGCTTGTTG
Pr 4^a^	TGTAAAACGACGGCCAGTGGTAACCCTGGATGTCCTGA	ATCCCGTTACAAATGGGACA
Pr 7^a^	TGTAAAACGACGGCCAGTAACGTACTGACAAACCAAACCA	AATTTTTCTTAGATTGCTAGGTTG
Pr 11^a^	TGTAAAACGACGGCCAGTCAAGCCAAATAAACGCATCC	TAGCGAAGAACACCAACGTG
Pr 12^a^	TGTAAAACGACGGCCAGTTCTTTAGTAGTTGCTTTGCTTGAA	AACATCTTGGCACCCCTTTA
Pr 16^a^	TGTAAAACGACGGCCAGTGGCAGGAACAAAAATTAAGCA	CGTTCCAAAGCGTTTTATGG
Pr 17^b^	TGTAAAACGACGGCCAGTCACACACTTGCTCCATGGTC	AAACTAGATAGCGAAAAA
Pr 18^b^	TGTAAAACGACGGCCAGTAAAGAAAGCTTCGTTTTAACGTG	CATTATCCACCCCCAAATCA
Pr 19^b^	TGTAAAACGACGGCCAGTACGACCCAATCCGTTGATAG	CCAAGGCACGTTAGAAGAAA

Forward primers all begin with the M13(−21) sequence (first 18 base pairs), allowing binding with a fluorescently labelled (6FAM or HEX) M13 primer (Schuelke, 2000).

a Reported in Mouton *et al*. (2007).

b Reported in Andras and Ebert (2013).

### Genetic analyses

We identified *P. ramosa* genotypes and analysed population structure with the Poppr package version 2.9.3 (Kamvar *et al*., 2014) in R version 4.2.2 (R Core Team, 2020). We excluded 1 locus (Pr17) from analyses because it was uniform across hosts. We filtered our dataset to include samples that amplified at eight or more out of 10 loci. This resulted in 93 samples that were used for the analyses (and between one and eight samples from a given species/lake/sampling date; Table 3). Multilocus genotypes (MLGs) were identified as samples that were identical at all loci (loci that did not amplify were assumed to be null alleles since several of these reactions were redone and yielded the same result). A distance matrix between the MLGs was constructed using Prevosti distance, which is the fraction of allelic differences between two samples out of all loci (Wright, 1978). To show relationships among genotypes, we built a dendrogram from the distance matrix using the unweighted pair group method with arithmetic mean (Sokal and Michener, 1958). We used bootstrapping, sampling 100 times to evaluate support for tree topology. Nine samples amplified two or more alleles for at least one locus. This most likely indicates that a single host was coinfected by multiple *P. ramosa* genotypes. As a result, we analysed two datasets: in one, we included the alleles with the highest amplification in each sample (i.e. ignoring coinfection, but using the dominant genotype within a host) and, in the other, we included all alleles found within an animal (i.e. including all coinfecting genotypes). We conducted the same analyses on both datasets. Results of the analyses on the two datasets were qualitatively similar. Here, we report results of analyses from the dataset without coinfections.

**Table 3.** Sample sizes for each lake, host and date

Lake	Date	Sampling round	Host	*N*
Bishop	8/4/2015	2	*Ceriodaphnia dubia*	1
			*Daphnia dentifera*	8
			*Daphnia retrocurva*	1
	8/17/2015	3	*D. dentifera*	1
	8/31/2015	4	*D. dentifera*	4
			*D. retrocurva*	4
	10/28/2015	8	*D. retrocurva*	2
Cedar	7/30/2015	2	*D. dentifera*	3
	11/6/2015	9	*D. dentifera*	8
Crooked W	10/9/2015	7	*D. dentifera*	8
			*D. retrocurva*	1
Gosling	9/1/2015	4	*D. dentifera*	2
Little Appleton	7/21/2015	1	*D. dentifera*	7
	9/30/2015	6	*D. dentifera*	7
Mill	8/7/2015	2	*Daphnia parvula*	1
	8/24/2015	4	*D. retrocurva*	7
			*D. parvula*	7
	9/8/2015	5	*D. retrocurva*	1
	9/21/2015	6	*D. retrocurva*	5
North	9/9/2015	5	*D. retrocurva*	7
			*D. parvula*	1
	10/21/2015	8	*D. retrocurva*	3
Walsh	8/7/2015	2	*D. dentifera*	2
			*D. retrocurva*	2

We analysed our data with Mantel tests and partial Mantel tests with R package ‘vegan’ version 2.6.4 (Oksanen *et al*., 2023) to understand how the Prevosti distance between every pair of parasite samples correlated with collection from the same *vs* different lake, the same *vs* different host species and number of days between collection.

We also carried out two hierarchical analyses of molecular variance (AMOVA) to quantify the extent to which parasite genotypes clustered by lake, host species and sample date (Excoffier *et al*., 1992). In the first AMOVA, host species was nested within lake; in the second, lake was nested within host species. We carried out the analyses both ways because neither lake nor host species is clearly a higher hierarchical level. Sample date was the third hierarchical level in both cases. This level was included to incorporate potentially important temporal information. However, small sample sizes at each sampling date make results from this level difficult to interpret. AMOVA partitions variation in Prevosti distances between samples into the hierarchical groups (Excoffier *et al*., 1992). We then randomly permuted the distance matrix 1000 times, each time calculating variance assigned to hierarchical groups to create a null distribution with which to test significance of population structure (Excoffier *et al*., 1992).

Finally, we calculated the fixation index (*F*_ST_) between the groups of *P. ramosa* samples from *D. dentifera* and *D. retrocurva* hosts to better understand structure within host species. *F*_ST_ was calculated using R package ‘hierfstat’ version 0.5.11 (Goudet, 2005). To test for isolation by distance we used linear models to determine if *F*_ST_/(1 − *F*_ST_) correlated with the natural log of geographic distance between lakes (kilometers) plus 1 or the amount of time between sampling dates (Rousset, 1997).

## Results

### *Pasteuria ramosa* infected multiple host species, but often at different times

**Figure 1.**
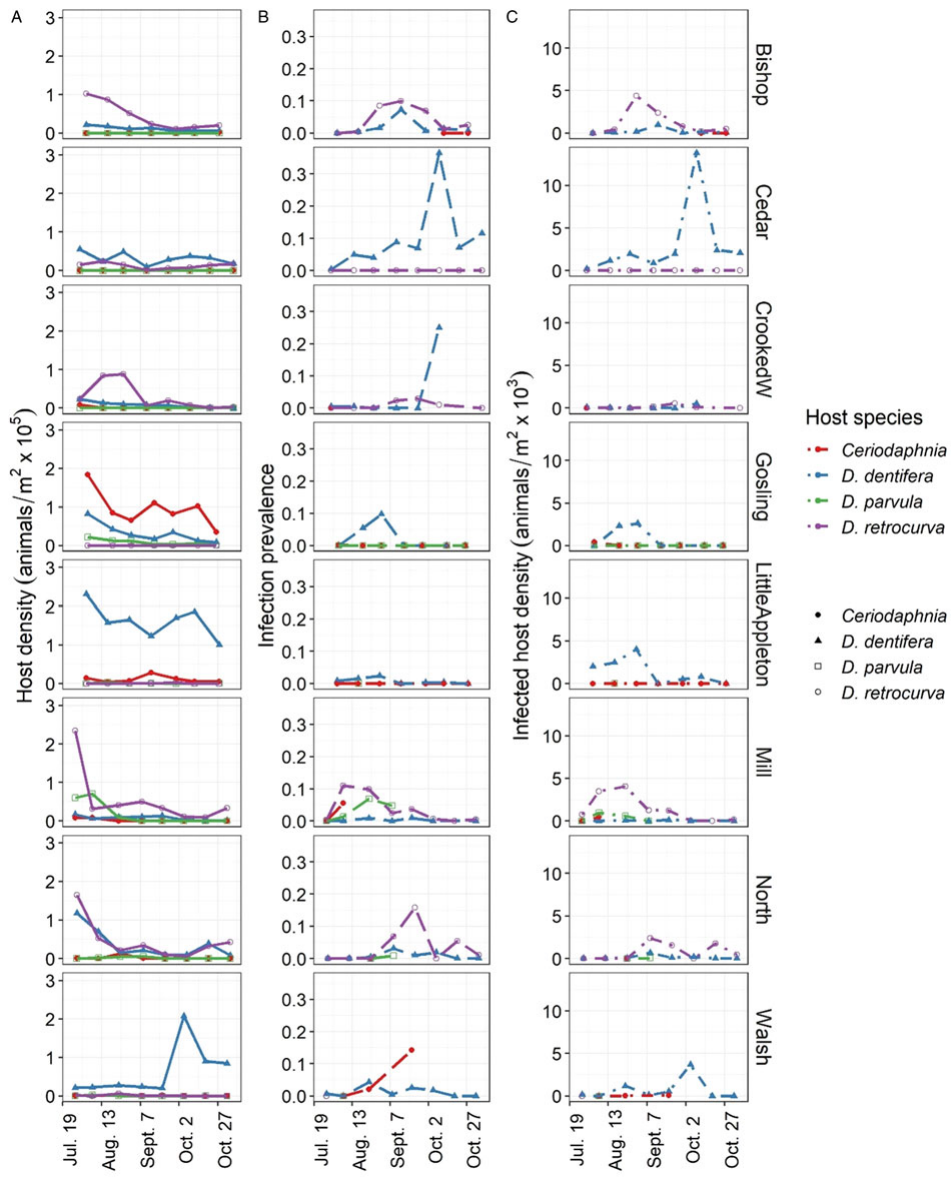
Lakes differed in host density and community composition (A) as well as prevalence (B) and (C) density of *P. ramosa-*infected hosts during the 2015 epidemic season (July–October). In (B) and (C), lines that terminate before the end of the sampling period indicate that too few hosts were counted (<20) to accurately assess prevalence of infection.

**Figure 2.**
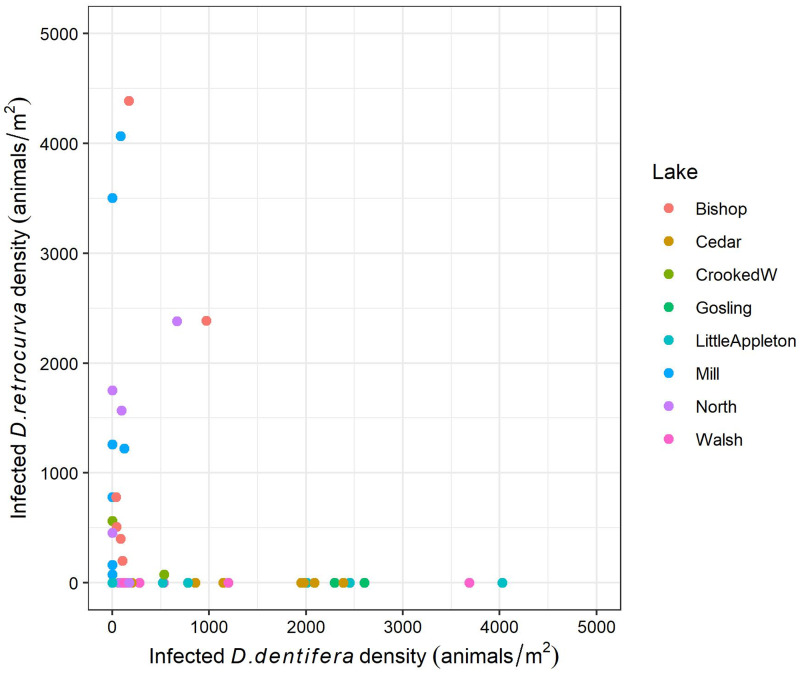
*Pasteuria ramosa* did not tend to infect *D. dentifera* and *D. retrocurva* in the same lakes at the same time. Each point is one sample date in one lake. For comparison purposes, this figure has the same scale on *x* and *y* axes. However, one datapoint had to be removed with this scaling: Cedar (10/09/2020) with 13 820 infected *D. dentifera* per m^2^ and 0 infected *D. retrocurva* per m^2^.

Lakes differed in host community composition during the epidemic season (July–October; Fig. 1A). *Pasteuria ramosa*-infected hosts were documented in all eight lakes and in four host species, *D. dentifera*, *D. retrocurva*, *D. parvula* and *C. dubia*. *Daphnia dentifera* and/or *D. retrocurva* were the most common species in seven of the eight lakes (Fig. 1A) and were the most parasitized (Fig. 1B and C). However, these hosts tended not to be infected at the same time in a given lake (Fig. 2).

### Genetic distance between *P. ramosa* samples tended to be smaller for samples from the same lake, the same host species and closer in time

In 93 *P. ramosa* samples, we detected between 3 and 14 alleles at each of 10 loci (mean: 9 alleles per locus). We identified 42 distinct MLGs based on the combination of alleles at the 10 loci.

**Figure 3.**
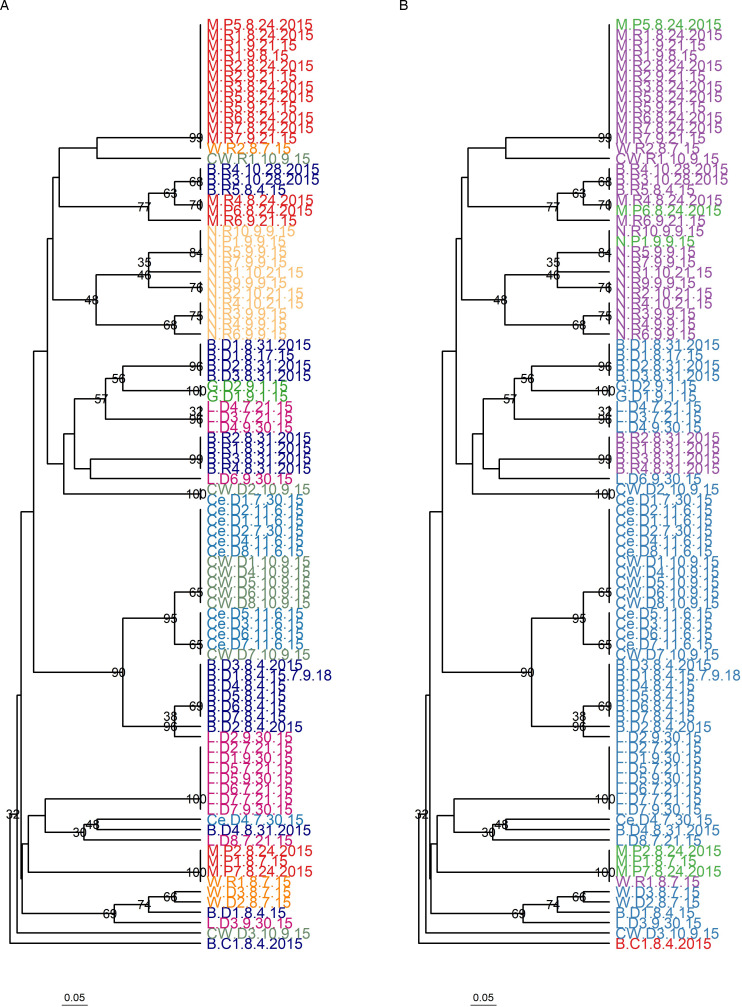
In natural outbreaks, *P. ramosa* strains clustered by lake, host species and by sampling date. (A) Dendrogram of *P. ramosa* isolates coloured by lake. (B) Dendrogram of *P. ramosa* isolates coloured by host species (purple: D. retrocurva*, blue: *D. dentifera*, green: Daphnia parvula*, red: *Ceriodaphnia*). Samples are named with the scheme: LakeCode.SpeciesCodeSampleNumber.SampleDate. Lake codes are M, Mill Lake; CW, Crooked Lake (Waterloo); B, Bishop Lake; L, Little Appleton Lake; G, Gosling Lake; Ce, Cedar Lake; N, North Lake; W, Walsh Lake. Species codes are R, *D. retrocurva*; D, *D. dentifera*; P, *D. parvula*. *D. retrocurva and *D. parvula* are sister species (Colbourne and Hebert, 1996). Bootstrap support above 30% is shown on nodes.

**Figure 4.**
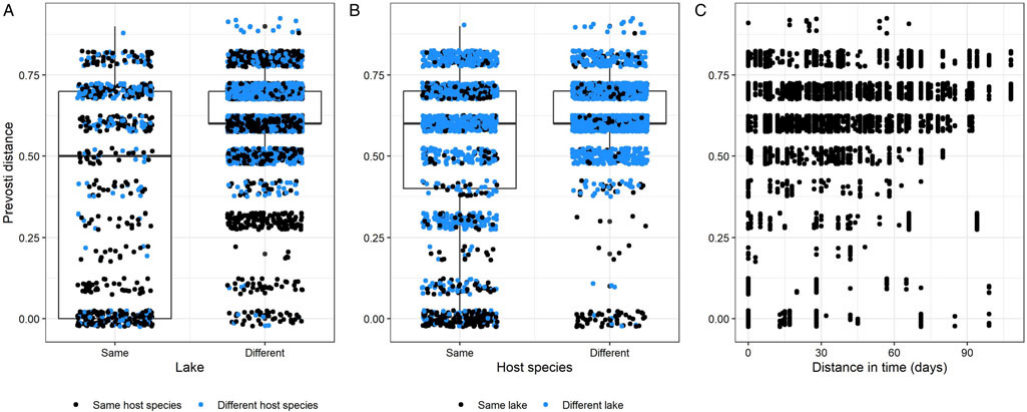
Lake, host species and time between sample collections are correlated with Prevosti distance between *P. ramosa* samples. (A) Prevosti distance between samples is greater if samples were collected from different lakes or (B) different host species. (C) Prevosti distance increased as samples were collected farther apart in time.

There was clear structuring of *P. ramosa* at the level of lake and host species (Fig. 3), and both Mantel tests and AMOVA supported lake, host species and sample date as important factors explaining *P. ramosa* genetic structure. Moreover, Mantel tests identified significantly higher Prevosti distances between *P. ramosa* samples from different lakes (*r* = 0.396, *P* = 0.001, Fig. 4A) and different host species (*r* = 0.237, *P* = 0.001; Fig. 4B). *Pasteuria ramosa* samples were also increasingly distinct with more time between sampling dates (*r* = 0.168, *P* = 0.001; Fig. 4C). Partial Mantel tests controlling for the effect of time between sampling dates gave similar impacts of same *vs* different lake (*r* = 0.372, *P* = 0.001) and same *vs* different host species (*r* = 0.248, *P* = 0.001) on Prevosti distances.

Our first AMOVA indicated marginally significant differences between *P. ramosa* genotypes in different lakes (variation explained = 11.9%, *P* = 0.085; Table 4), significant differences between genotypes infecting different host species within lakes (variation explained = 24.0%, *P* = 0.025; Table 4), and significant differences in *P. ramosa* genetic structure over time during outbreaks (variation explained = 15.2%, *P* = 0.002; Table 4). The same genotypes were identified in the nearby lakes Crooked (W) and Cedar as well as in Walsh and Mill (Fig. 3A), perhaps explaining the marginal effect of lake as the highest level of hierarchy in this analysis. Our second AMOVA treated host species as the highest level of hierarchy and lake and sample date as lower levels. In this analysis, host species was a marginally significant factor explaining the variation of *P. ramosa* genotypes (variation explained = 7.01%, *P* = 0.067; Table 4); within species, lakes were a significant factor explaining variation (variation explained = 29.97%, *P* = 0.002; Table 4); sampling date within species/lake was also a significant factor explaining variation (variation explained = 14.88%, *P* = 0.001; Table 4). The lack of significance of host species on parasite genetic structure in the second AMOVA can potentially be explained by finding the same parasite genotypes infecting sister species, *D. retrocurva* and *D. parvula*, in Mill Lake (Fig. 3B).

**Table 4.** Hierarchical analysis of variance organizing parasite samples by two hierarchical regimes. AMOVA 1 designates lake as the highest level followed by host species and sample date. AMOVA 2 designates host species as the highest level followed by lake and sample date

Variance component	Variance	% total	*P*^a^	*ϕ*-statistics
AMOVA 1: Lakes species as highest level of hierarchy				
Between lakes	0.94	11.90	(Greater) 0.085	*ϕ*_Lake−Total_ = 0.12
Between host species	1.88	23.96	(Greater) 0.025	*ϕ*_Host−Lake_ = 0.27
Between dates	1.19	15.15	(Greater) 0.002	*ϕ*_Date−Host_ = 0.24
Within dates	3.85	49.00	(Less) 0.001	*ϕ*_Date−Total_ = 0.51
AMOVA 2: Host species as highest level of hierarchy				
Between host species	0.56	7.01	(Greater) 0.067	*ϕ*_Host−Total_ = 0.07
Between lakes	2.40	29.97	(Greater) 0.002	*ϕ*_Lake−Host_ = 0.32
Between dates	1.19	14.88	(Greater) 0.001	*ϕ*_Date−Lake_ = 0.24
Within dates	3.85	48.13	(Less) 0.001	*ϕ*_Date−Total_ = 0.52

a The *P* values are calculated by 1000 random permutations of the distance matrix (composed of Prevosti distances) between genotyped parasites. Significance is attained if the observed *ϕ*-statistic (and variance component) is greater or smaller than it would be by chance (Excoffier *et al*., 1992).

### Isolation by distance for *P. ramosa* from one host species but not for another

**Figure 5.**
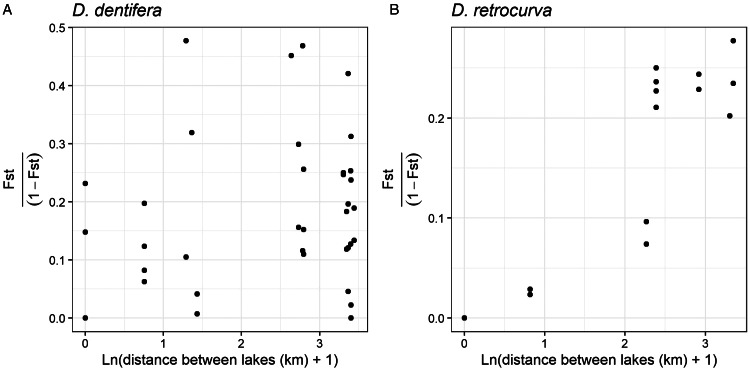
*F*_ST_/(1 − *F*_ST_) values between *P. ramosa* groups infecting *D. dentifera* and *D. retrocurva* showed different relationships with the geographic distance between collection lakes. (A) *F*_ST_/(1 − *F*_ST_) values between *P. ramosa* groups from *D. dentifera* hosts did not show any relationship with geographic distance between the lakes, while (B) *F*_ST_/(1 − *F*_ST_) values between *P. ramosa* groups from *D. retrocurva* hosts showed a significant positive relationship with geographic distance between the lakes.

When comparing *P. ramosa* from one host species across lake-days, *F*_ST_ within *D. dentifera* hosts ranged from 0 to 0.323 and from 0 to 0.217 for samples from *D. retrocurva*. Within *D. dentifera*, *F*_ST_/(1 − *F*_ST_) did not reveal a pattern of isolation by geographic distance between lakes (*F* = 0.86, *P* = 0.36; Fig. 5A) or a correlation between the amount of time between sampling and genetic divergence (*F* = 1.75, *P* = 0.20). However, within *D. retrocurva*, *F*_ST_/(1 − *F*_ST_) correlated with geographic distance between lakes (*F* = 49.2, *P* < 0.001; Fig. 5B), indicating isolation by distance, but not with the amount of time between sampling (*F* = 1.08, *P* = 0.32).

### Lake and host species as important levels of *P. ramosa* structuring

Overall, there is support for both lake and host species as important levels of structuring of parasite populations. However, it is unclear which level (lake or host species) is more important. Few lakes had large numbers of *P. ramosa* samples from multiple host species, which could be the cause of this lingering uncertainty – a result which, on its own, suggests that host species is an important driver of patterns of infection (as also suggested in Fig. 2).

## Discussion

In this study, we asked how the common parasite *P. ramosa* navigates complex environments with multiple host species distributed across lakes and where each lake has a spore bank. Importantly, these communities differ in host species composition. Mirroring this, we found that *P. ramosa* outbreaks differed in size and which host species were parasitized: while multiple species were infected, infections tended to be common in only one host species in a given lake. Consistent with this, we found that *P. ramosa* structured by host species, especially within lakes, and that lake also defined structure, especially when host species was treated as the highest level of hierarchy. We also found that *P. ramosa* structured by sampling date within lakes and host species, supporting other studies that suggest that *P. ramosa* evolves through epidemics.

We predicted that *P. ramosa* populations would show structuring between lakes for two reasons. First, *P. ramosa* genotypes are host genotype specific due to variation in host and parasite proteins that allow for attachment of the bacterium to cells in the host oesophagus, the initial site of infection (Duneau *et al*., 2011; Routtu and Ebert, 2015; Bento *et al*., 2017). Theory predicts that host-specific parasites are more likely to be locally adapted and differentiated between populations (Barrett *et al*., 2008). Second, *P. ramosa* spores can survive for decades in sediments (Decaestecker *et al*., 2007), thus lakes may differ in *P. ramosa* standing diversity due to evolution during historical outbreaks (Andras *et al*., 2018) and stochasticity in parasite survival and sampling from the spore bank. Our prediction that *P. ramosa* would structure by lakes was generally supported by our Mantel tests and AMOVA analyses. It was interesting to us that the geographic distance between lakes was associated with increasing *F*_ST_/(1 − *F*_ST_) between groups within *D. retrocurva* but not within *D. dentifera*. Similar to our finding for *D. dentifera*, a previous study on European *P. ramosa* that analysed infection phenotypes of more than 50 isolates did not find correlations between infectivity and geographic distance (Fredericksen *et al*., 2021). In our study, the same parasite genotypes were found in different lakes: genotypes were shared between Walsh and Mill lakes (in *D. retrocurva* and *D. parvula*) and in Cedar and Crooked (W) lakes (in *D. dentifera*). Walsh, Mill, Cedar and Crooked (W) lakes are all within 2.7 km of each other in the Waterloo State Recreation Area. We also found the same parasite genotypes across lakes in a different study which analysed samples collected in a different year (Shaw and Duffy, 2023). It is possible that recreational use of these lakes as well as transport by waterfowl (Green and Figuerola, 2005) could move parasites between lakes. Alternatively, these samples could, in fact, be different from each other at loci beyond the ones used in this study. Sequencing *P. ramosa* samples at barcoding loci – such as the hypervariable region that has recently been found to govern infectivity (Andras *et al*., 2020) – might allow for finer resolution of patterns of diversity within and across lakes.

We found that *P. ramosa* did not readily infect across certain species barriers but did across others. Previous research has shown that *P. ramosa* spores can attach to multiple host species' oesophagi (Duneau *et al*., 2011; Luijckx *et al*., 2014) despite genotype specificity of infection success within species, indicating that different host species may share resistance and susceptibility alleles. However, in previous studies, the same parasite genotype rarely infected two host species (despite attachment) indicating that additional steps in the hosts' resistance pathways operate differently in different host species (Luijckx *et al*., 2014). Here, we found evidence that *P. ramosa* strains could move between closely related host species, *D. retrocurva* and *D. parvula*. These species are sister to each other and separated by less than 50 000 years of evolution (Costanzo and Taylor, 2010). If closely related hosts offer a more similar within-host environment or resistance pathway, this close phylogenetic relationship may explain how the same parasite genotype is able to exploit both; however, it is also clear that there can be substantial variation in interactions with parasites among genotypes of the same species (Duffy and Sivars-Becker, 2007; Auld *et al*., 2012; Pfenning-Butterworth *et al*., 2023). In the lab, we have been successful at infecting *D. parvula* with spores from *D. retrocurva*, and we have moved *P. ramosa* between another closely related and hybridizing pair, *D. dentifera* and *D. mendotae* (C. D. Gowler and C. L. Shaw, unpublished data). In contrast, we did not observe the same *P. ramosa* genotypes in *D. dentifera* and *D. retrocurva*, and the genotypes infecting these two host species tend to differ at a number of loci. This pattern suggests that the parasite is less likely to move between these two hosts, at least under the conditions we observed. Again, more in-depth sequencing efforts would be useful to determine how distinct these parasite genotypes are and to estimate for how long they have been evolving separately.

Our study presents evidence that *P. ramosa* could evolve over the course of outbreaks, which supports earlier findings (Gowler *et al*., 2022; Shaw and Duffy, 2023). However, due to the small sample sizes at different sampling dates for each lake and host species in our study, these results could also be due to sampling different genotypes at different sampling dates by chance. If our results do reflect evolutionary processes, selection on parasite populations could be driven by shifts or cycling in host community structure due to parasitism (Duncan and Little, 2007) or other factors (Hu and Tessier, 1995; Geedey *et al*., 1996). Parasite evolution could also be in response to selection from non-host associated factors (e.g. abiotic conditions; Mitchell *et al*., 2005; Vale and Little, 2009; Rogalski and Duffy, 2020). Future studies that explore how host community structure influences parasite evolution would be particularly helpful for understanding the evolution of multi-host parasites.

The patterns we documented raise several questions about disease dynamics and feedbacks in this system. We are curious about how host species composition impacts epidemic size and if it is possible to predict which host species will be more severely impacted. *P. ramosa* appears to parasitize the more common host, but, in some cases, epidemics occur primarily in one host species even when multiple are comparable in density (e.g. North Lake, Bishop Lake and Cedar Lake in this study). It is possible that hosts destroy spores that are not capable of infecting them since spores become activated during contact with a potential host and then subsequently have a lifespan of only about 24 h (Ebert *et al*., 2016). Spore destruction by non-susceptible hosts could lead to the ‘friendly competition’ phenomenon that occurs between host species with a fungal parasite in *Daphnia* hosts (Hall *et al*., 2009). A recent study found that different host species can reduce the infectivity of spores in a focal host species in the lab but did not find evidence of a dilution effect in the field (Fearon *et al*., 2023). Nonetheless, feedbacks across years could occur if spore banks are seeded with *P. ramosa* spores that are infective to one host but not its main competitor. Investigating host species structure, epidemic dynamics and which hosts are predominantly infected across years would be fruitful for understanding if epidemics can have consequences for community composition and parasitism in future years.

We quantified the genetic structure of the parasite, *P. ramosa*, in infected hosts during natural outbreaks across lakes, host species and over time within outbreaks. We found that parasites structure among lakes, host species and sample dates indicating that there are barriers to parasite establishment between lakes and certain host species in this system. However, transmission between lakes may occur due to transport of spores by wildlife or humans, and transmission between host species appears to occur when host species are closely related phylogenetically. We also found changes in parasite structure over time, supporting other studies that have documented evolution within outbreaks. Thus, analyses of parasite genetic structure can implicate ecological and evolutionary forces acting on parasites. Additional studies documenting parasite population structure across natural host–parasite systems could yield patterns that help predict parasite outbreaks in complex environments.

## Data Availability

The data and R code used for analyses presented here are available at https://github.com/clarashaw/Pasteuria2015/tree/main.
